# In Situ Fenestration and Carotid-Subclavian Bypass for Left Subclavian Artery Revascularization During Thoracic Endovascular Aortic Repair

**DOI:** 10.1007/s00270-024-03675-3

**Published:** 2024-03-15

**Authors:** Bowen Fan, Kun Fang, Chuan Tian, Jie Fang, Dong Chen, Jiawei Zhao, Mingyao Luo, Chang Shu

**Affiliations:** 1 State Key Laboratory of Cardiovascular Disease, Center of Vascular Surgery, Fuwai Hospital, National Center for Cardiovascular Disease, Chinese Academy of Medical Sciences and Peking Union Medical College, Beijing, 100037 China; 2 Department of Vascular Surgery, The 2nd Xiangya Hospital of Central South University, Changsha, 410011 China; 3 Department of Vascular Surgery, Central-China Branch of National Center for Cardiovascular Diseases, Henan Cardiovascular Disease Center, Fuwai Central-China Hospital, Central China Fuwai Hospital of Zhengzhou University, Zhengzhou, 450046 China; 4 Department of Vascular Surgery, Fuwai Yunnan Cardiovascular Hospital, Affiliated Cardiovascular Hospital of Kunming Medical University, Kunming, 650102 China

**Keywords:** Aortic arch, Thoracic endovascular aortic repair, Thoracic stent-graft, Carotid-subclavian bypass, In situ needle fenestration

## Abstract

**Purpose:**

To evaluate the safety and feasibility of left subclavian artery (LSA) revascularization techniques during thoracic endovascular aortic repair (TEVAR)—the in situ needle fenestration (ISNF) technique and the carotid-subclavian bypass (CS-Bp)—for complicated aortic pathologies.

**Methods:**

A retrospective single-center observational study was conducted to identify all patients with thoracic aortic pathologies who underwent TEVAR with LSA revascularization using either CS-Bp or ISNFs from January 2014 to December 2020.

**Results:**

One hundred and twelve consecutive patients who received TEVAR with LSA revascularization were included. Among them, 69 received CS-Bp and 43 received ISNF (29 using the Futhrough adjustable puncture needles, 14 using the binding stent-graft puncture systems). Technical success, defined as achieving aortic arch pathology exclusion and LSA preservation, was attained in 99.1% patients. Early mortality was 0.9%. Major adverse events within 30 days, including one cerebral hemorrhage, one cervical incision hemorrhage, one stroke and two paraplegia, were exclusively observed in the CS-Bp group. Immediate type I, II and III endoleaks occurred in 0%, 4.7% and 2.3% in the ISNF group, respectively, compared to 0%, 2.9% and 0% in the CS-Bp group.One hundred and eight (97.2%) patients were available for follow-up at a median 50 (maiximum of 103) months, revealing a LSA patency rates of 99.1%. Six patients died during follow-ups—five in the CS-Bp group and one in the ISNF group. Cause of death include one aortic-related stent-graft infection, three non-related and two with unknow causes. The survival exhibited no significantly different between the ISNF (97.7%) and CS-Bp (89.9%) groups (*p* = 0.22).

**Conclusions:**

Both CS-Bp and ISNF are feasible techniques for LSA reconstruction in TEVAR. ISNF, whether using Futhrough or BPS, seems to be competitive with CS-Bp.

**Graphical Abstract:**

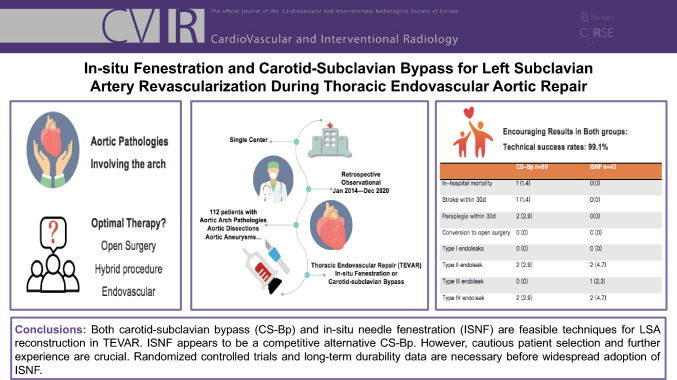

## Introduction

Thoracic endovascular aortic repair (TEVAR) has become the first-line therapy for descending aortic diseases [1]. However, the optimal approach for patients with aortic arch pathologies, involving the left subclavian artery (Zone 2), remains controversial. While conventional surgical repair remains the gold standard for addressing aneurysms or dissections in the aortic arch, the associated extensive surgical trauma poses a notable risk of complications [2, [3]. Hybrid aortic repair, involving procedures like carotid-subclavian bypass (CS-Bp) or transposition combined with TEVAR, offers a less invasive alternative to conventional surgical repair. This method serves to extend the proximal landing zone for TEVAR. Although studies have reports favorable outcomes with the use of CS-Bp in TEVAR, attention must be paid to the associated complications, including early mortality, paraplegia and nerve injuries [4–7].

Various TEVAR-assisted approaches, such as chimney, in situ fenestration, fenestrated and branched stent-graft, have emerged as viable options for the repair of aortic arch pathologies through a completely endovascular approach [6–13]. Among these techniques, in situ fenestration, encompassing in situ needle fenestration (ISNF) and energy-based (radiofrequency or laser) in situ fenestration, stands out as a potential reasonable method. This technique involves performing in situ fenestrations after TEVAR, followed by the deployment of bridge stents to preserve branch vessels [14]. Encouraging outcomes of in situ fenestration have been reported in several studies [15–21].

The objective of this study is to examine and compare the perioperative and mid-term outcomes of aortic arch pathologies requiring LSA revascularization during TEVAR, utilizing either ISNF or CS-Bp within a single center and focused on a selected cohort of patients.

## Method

### Study Design and Patient Eligibility

A retrospective single-center observational study was conducted to identify consecutive patients presenting with thoracic aortic pathologies, encompassing aortic dissections, aneurysms, and penetrating ulcers from January 2014 to December 2020. The study focused on patients necessitating LSA revascularization due to hostile proximal landing zones. These zones were defined as either having healthy landing zones measuring less than 1.5 cm or exhibiting excessive tortuosity, or the presence of calcifications. Exclusion criteria are as follows: 1. Presence of a more than 1.5 cm healthy segment of the aorta suitable for sufficient fixation and sealing; 2. the aortic pathologies involved the left common carotid artery and/or the innominate artery that required revascularization of multiple supra-aortic branches; 3. patients with contraindications for surgical procedures; and 4. patient refusal to provide informed consent.

**Fig. 1:**
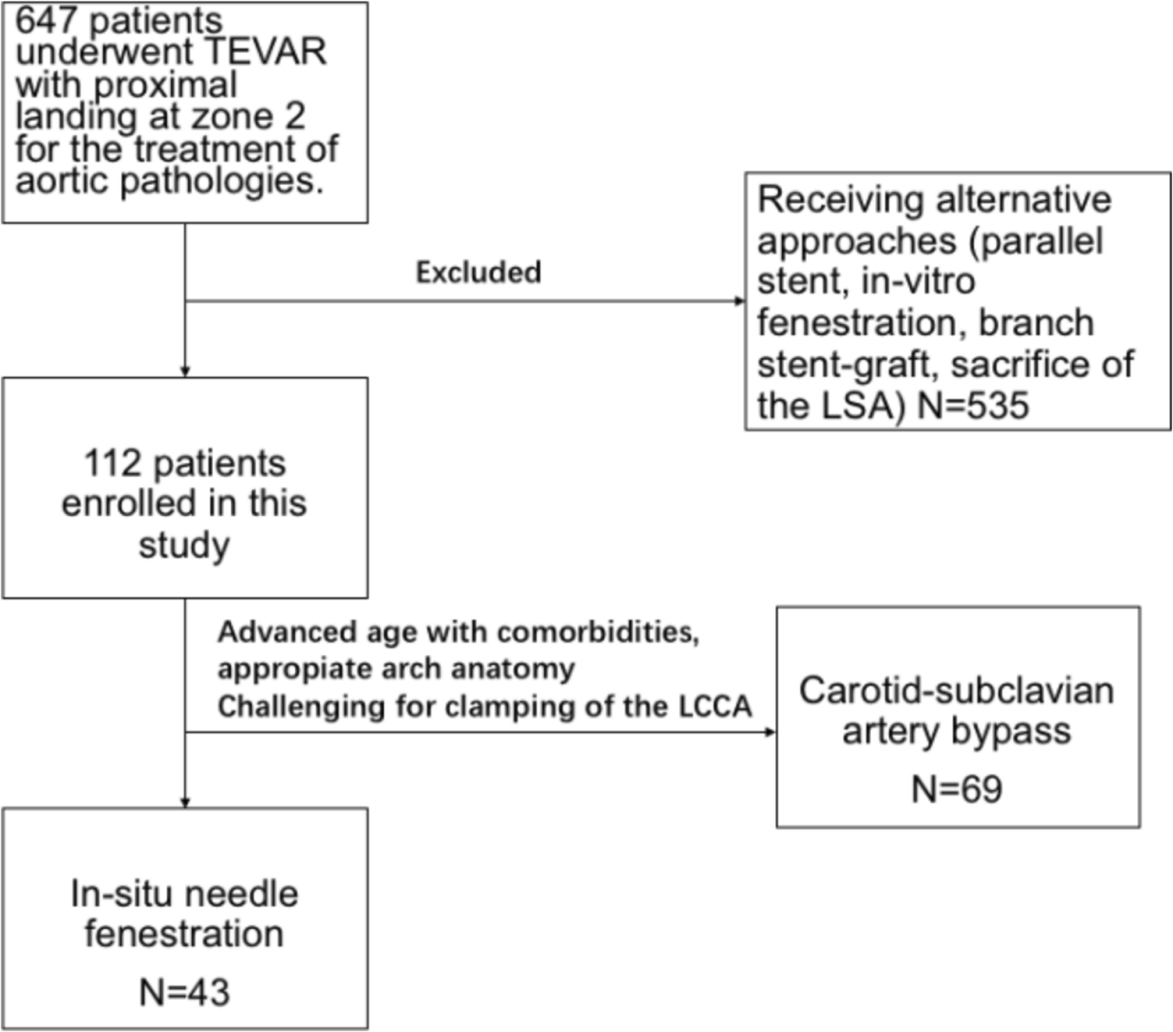
The patient flow chart

CS-Bp was the preferred method for LSA revascularization in accordance with the preveiling guidelines. ISNF was employed in specific cases: (a) patients exhibited advanced age and comorbidities favoring total endovascular procedures. (b) Preoperative cerebral CT and cervical ultrasound indicate potential risk associated with CS-Bp procedure due to the clamping of LCCA and (c) suitable anatomy was present, aortic pathologies on the outer curvature of the arch close to (< 15 mm) or involving the LSA, with a ≥ 45° take-off angle of the LSA from the arch and without significant tortuosity and calcification of the aorta and the LSA. The patient flow chart is illustrated in Fig. 1.

The institutional review boards of our center approved the study, and informed consent was obtained from all the patients and their relatives (Table 1).

**Table 1:** Baseline characteristics of patients

	ISNF (*n* = 43)	CS-Bp (*n* = 69)	*p*
Male	35 (81.4)	63 (91.3)	0.068
Age, mean (SD)	64.4 (9.7)	56.4 (13.2)	0.001
Timing of treatment			
Elective (> 2 weeks)	24 (55.8)	37 (53.6)	0.82
Emergency (< 2 weeks)	19 (44.2)	32 (46.4)	0.82
Main Diagnosis			
Aortic dissection	20 (46.5)	31 (44.9)	0.87
Aortic aneurysm	11 (25.6)	19 (27.5)	0.82
Penetrating aortic ulcer	10 (23.3)	17 (24.6)	0.868
Intramural hematoma	2 (4.7)	1(1.4)	0.307
Post-TEVAR complication	0 (0)	1 (1.4)	0.428
Clinical presentation			
Chest and/or back pain	20 (46.5)	32 (46.4)	0.989
Abdominal pain	7 (16.3)	12 (17.3)	0.879
Comorbidities			
Hypertension	43 (100.0)	68 (98.6)	0.428
Coronary heart disease	18 (41.9)	21 (30.4)	0.217
Diabetes mellitus	13 (30.2)	19 (27.5)	0.759
COPD	4 (9.3)	4 (5.8)	0.484
Previous Stroke	7 (16.3)	6 (8.7)	0.223

Values are *n* (%) except that age mean (SD)

*ISNF* in situ needle fenestration, *CS-Bp* carotid-subclavian bypass, *COPD* chronic obstructive pulmonary diseases

### Preoperation Evaluations

An interdisciplinary board, comprising endovascular and cardiovascular surgeons, neurologists, radiologists and anaesthesiologists, collaborated to holistically assess each patient and determine the most appropriate treatment modalities. All procedures were performed under general anesthesia in a hybrid operating suite.

### Carotid-Subclavian Bypass

The CS-Bp procedure was performed concurrently with TEVAR in one session. Surgical exposure of the LSA and LCCA was achieved through a supraclavicular incision. The prosthetic graft, Hemashield (Maquet; Rastatt; Germany) or Goretex (Gore; Flagstaff; USA), was anastomosed end-to-side to the LSA distal to the origin of the left vertebral artery. Subsequently, it was tunneled beneath the internal jugular vein, before being anastomosed end-to-side to the LCCA. The proximal side of the LSA was ligated or coiled proximal to the orifice of the vertebral artery. Subsequently, TEVAR was performed.

The choice of thoracic stent-graft was at the surgeon’s discretion (Table 2), including Valiant (Medtronic; Minneapolis; USA), Ankura (Lifetech; Shenzhen; China), Zenith (Cook; Bloomington; USA), Hercules (MicroPort; Shanghai; China) or Relay (Terumo; Sunrise; FL; USA). The oversizing of the thoracic stent-graft generally ranged from 10% for aortic dissections and intramural hematomas to 20% for aneurysms and penetrating ulcers.

**Table 2:** Materials used in the 43 in situ needle fenestration procedures by lesion type

Thoracic aortic aneurysms/penetrating aortic ulcer	21
Ankura + Viabahn	7
Ankura + Express LD	9
Ankura + Fluency	2
Ankura + Express LD + Viabahn	1
Ankura + Dynamics	1
Valiant without bridge stent-graft	1
Type B aortic dissection	20
Ankura + Viabahn	10
Ankura + Express LD	6
Ankura + Medtronic branch stent	1
Valiant + Express LD	1
Zenith + Express LD	1
Ankura + Fluency (convert to chimney stent-graft)	1
Intramural hematoma	2
Ankura + Express LD	1
Ankura + Viabahn	1

### In Situ Fenestration Technique

Ankura thoracic aortic stent-graft was employed for all 43 patients undergoing INSF due to the assumed favorable characteristics of the expanded-polytetrafluoroethylene (e-PTFE) membrane in facilitating puncturing [22].

**Fig. 2:**
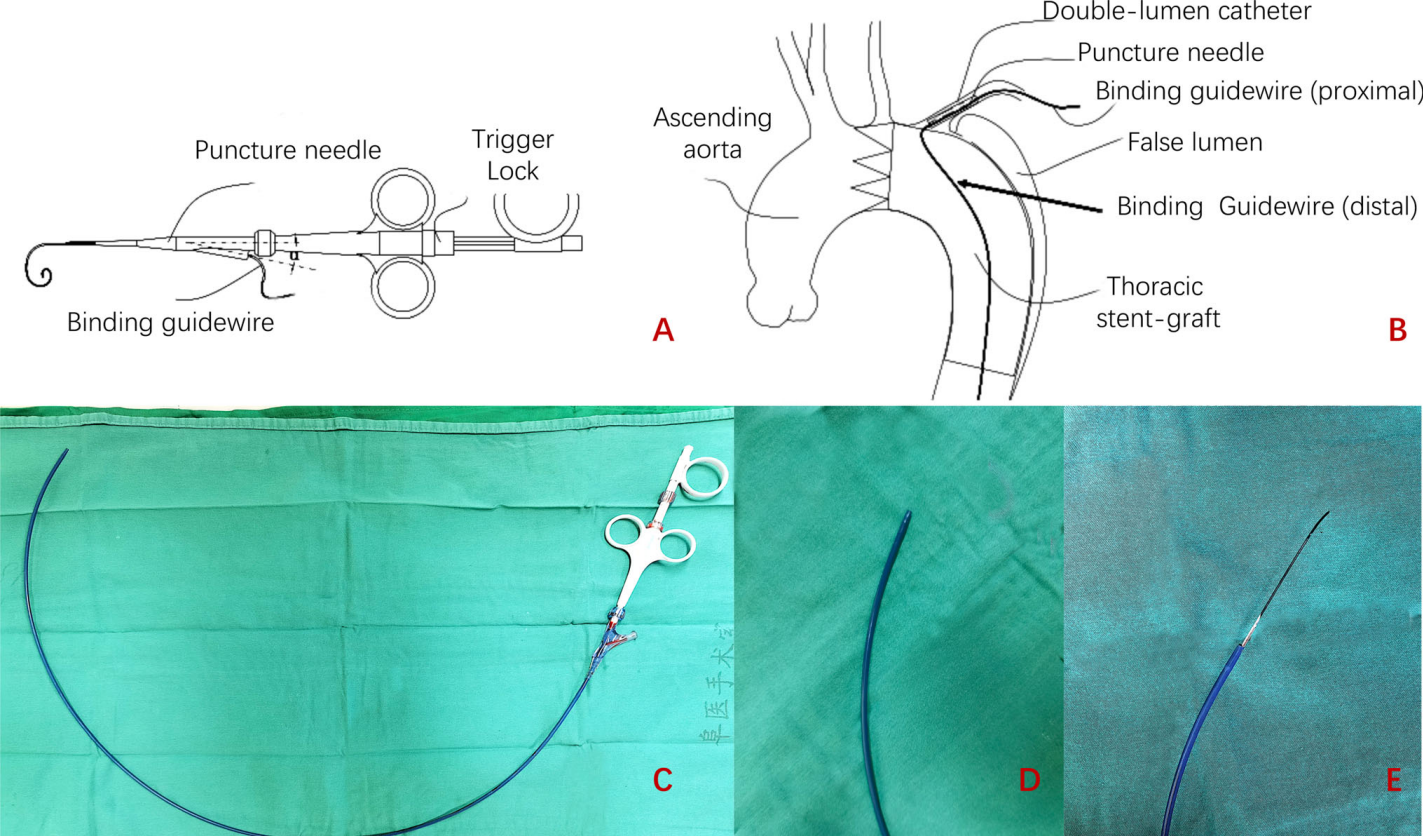
The Binding Stent-graft Puncture System (BPS). **A**, **B** The design of BPS. This device provides a double-lumen sheaths that allows advancement of a stiff guidewire toward the abdominal aorta for binding the puncture device in one lumen, and storing the puncture needle in the other lumen. **C** The figure of BPS used in an operation. **D**, **E** When the trigger was unlocked, the puncture needle could be activated by pushing the trigger. Immediately after the puncturing, a guidewire could be advanced through the puncture needle

Initially, the ISNF was established using an adjustable puncture needle (Futhrough™, Lifetech; Shenzhen; China), a technique previously described by Wang et al. [23]. Since 2019, the puncture device was transitioned to a binding stent-graft puncture system (BPS, Lifetech; Shenzhen; China). This device has a double-lumen sheaths allowing advancement of a pull-through guidewire toward the abdominal aorta in one lumen, while the pucture needle is stored in the other (Fig. 2). By applying tension on the pull-through stiff guidewire from the LSA to the abdominal aorta, the puncture system can be securely positioned for a precise and safe puncture procedure.

**Fig. 3:**
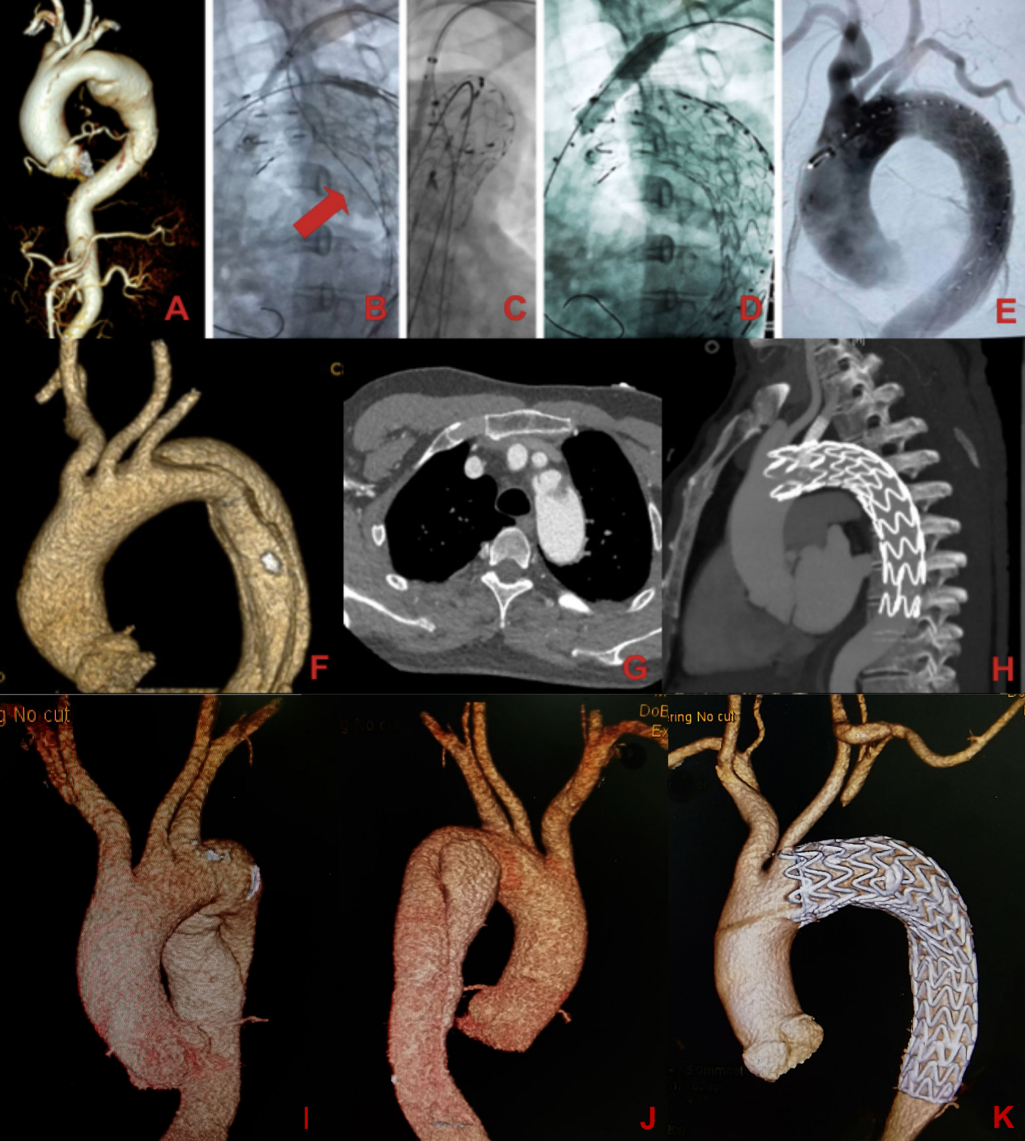
Cases in the study. **A**–**E** The binding stent-graft puncture system (BPS) in in situ needle fenestration (ISNF) to revascularize multiple aortic branches. **A** Preoperative CTA shows an aortic aneurysm with hostile landing zone for TEVAR. **B** The binding guidewire (red arrow) was advanced toward the abdominal aorta to bind the system firmly. **C** The thoracic stent-graft was punctured and the guidewire was advanced into the aortic lumen successfully. **D** A balloon-expandable branch stent was deployed with its proximal side in the aortic lumen, with its distal side in the branch. **E** The completion DSA shows ideal results. **F**, **G**, **H** Results after successful ISNF for a patient with aortic dissection. **I**, **J**, **K** Results after successful carotid-subclavian bypass with TEVAR for a patient with aortic dissection

The BPS was introduced from the left brachial access. Following the completion of TEVAR, the puncture procedure was meticulously activated until the position of the puncture device was repeatedly confirmed from various angles. This procedure created a small-sized fenestration through which a 0.018 or 0.035 guidewire was passed. The fenestration was then carefully dilated using a small-sized balloon (3–5 mm) and a branch stent-graft/stent was implanted into the LSA. If the aortic pathology was very close to (< 5 mm) or involved the LSA, a covered branch stent-graft was employed; otherwise uncovered balloon-expandable stents were used. The choice of specific bridging stents (Table 2), such as Viabahn (Gore; Flagstaff; USA), Fluency (Bard; NJ; USA), or Express LD (Boston Scientific; MA; USA), was left to the surgeon's discretion. Figure 3 illustrated the procedures and cases of ISNF. The use of Futhrough adjustable puncture needle was similar to BPS, with the distinction that Futhrough stabilized the puncture needle by inflating the balloon inside the LSA.

To avoid dislodgement, long reinforced sheaths (Fustar; Shenzhen; China) were used as the introduction system for the branch stent/stent-graft.

Balloon molding (Sterling PTA Balloon; Boston Scientific; MA; USA, Advance Low-profile PTA Balloon, Cook; Bloomington; USA) of bridging stents was consistently applied in all cases undergoing ISNF.

### Postoperative Management

Primary success was defined as endovascular exclusion of the aortic arch pathology and preservation of the LSA using either approaches, confirmed by both the completion DSA and the postoperative computed tomography angiography (CTA). All patients received postoperative management with OMT, including antiplatelet drugs, antihypertensive drugs and beta-blockers. Dual antiplatelet therapy was exclusively prescribed in the ISNF group. Follow-ups, including CTA scans and assessments of complications, were scheduled at 3, 6 and 12 months and annually thereafter. Early outcomes pertain to results within 30 days, while late outcomes extend beyond 6 months. Endoleaks and patency (no > 70% stenosis or occlusion) of supra-arch arteries were identified based on CTA findings.

### Statistical Analysis

Continuous data are expressed as the mean (± SD) or median (range). Cumulative survival was analyzed using Kaplan–Meier survival curves. Analyses were conducted using SPSS 23.0 software (IBM Corporation). GraphPad Prism 8 (GraphPad Software) was used for graphical representation of data.

## Results

One hundred and twelve patients were included. Sixty-nine patients undergo CS-Bp (mean age 64.4 ± 9.7 years; 63 men), and forty-three patients (mean age 64.4 ± 9.7 years; 35 men) were selected to undergo ISNF to preserve the LSA (29 using the Futhrough adjustable puncture needle, 14 using the binding stent-graft puncture system); Details of patients are listed in Table 1.

### Early Outcomes

Primary technical success was achieved in 111 cases (111/112, 99.1%). The single failure occurred in the early stage within the ISNF group and was subsequently converted to chimney technique.

Overall, the median operation time (from the beginning of surgical cutdown to the finish of suturing) was significantly longer in the CS-Bp group [166 min, range (80–311) vs 64 min, range (44–108), *p* < 0.001].

Early mortality was 0.9% (*n* = 1). In the CS-Bp group, two patients required mechanical ventilation for over 48 h, one due to cerebral hemorrhage resulting in death within 30 days, and the other experiencing preoperative malperfusion syndrome and pleural effusion requiring postoperative bedside hemodialysis and mechanical ventilation. There was no significant difference in mortality between the CS-Bp and ISNF groups (1.4% vs 0.0%, *p* > 0.05). Open conversion was required in neither groups. All patients were routinely transferred to the intensive care unit (ICU), where the length of stay ranged from 1 to 15 days (median 1; *p* > 0.05 between groups).

In the CS-Bp group, one patient (1.4%, 1/69) experienced cervical hemorrhage at the supraclavicular incision, necessitating surgical reintervention and blood transfusion within two days. One patient (1.4%) suffered from ipsilateral ischemia stroke. Two patients (2.9%, 2/69) developed paraplegias, including one temporary paraplegia (recovered one week later) and one persistent paraplegia, despite prophylactic drainage of cerebrospinal fluid and management of systolic blood pressure. There were no reported lymphatic or cervical nerve complications in the CS-Bp group.

No major adverse events were observed within 30 days in the ISNF group. The median hospitalization was 9 days in the ISNF group and 11 days in the CS-Bp group (*p* = 0.43). At discharge, 67 (59.8%) patients were prescribed a single antiplatelet drug. The 43 patients in ISNF group (38.4%) received dual antiplatelet therapy, and two (1.8%) were given oral anticoagulants.

Immediate early types I, II, III and IV endoleaks occurred in 0 (0%), 2 (4.7%), 1 (2.3%) and 2 (4.7%) in ISNF group, respectively, (immediate endoleak diagnosed by completion DSA, detailed in Table 3). Types I, II, III and IV endoleaks occurred in 0 (0%), 2 (2.9%), 0 (0%) and 2 (2.9%) in CS-Bp group, respectively.

**Table 3:** Periprocedural findings in 30d

	CS-Bp *n* = 69	ISNF *n* = 43	*P*
LSA revascularized	69 (100)	43 (100)	–
Operation time (minutes)	166 (80–311)	64 (44–108)	< 0.001
Fluoroscopic time (minutes)	20 (17–28)	28 (20–96)	< 0.001
Contrast volume (ml)	60 (40–110)	100 (80–120)	< 0.001
Blood loss (ml)	50 (30–80)	10 (5–30)	< 0.001
In-hospital mortality	1 (1.4)	0 (0)	0.733
Major adverse events in 30d			
Stroke	1 (1.4)	0 (0)	0.733
Paraplegia	2 (2.9)	0 (0)	0.644
Mechanical ventilation over 48 h	2 (2.9)	0 (0)	0.644
Cervical incision hemorrhage	1 (1.4)	0 (0)	0.733
Cranial and cervical nerve injury	0 (0)	0 (0)	–
Readmission/conversion to open surgery	0 (0)	0 (0)	–
Early endoleaks			
Type I endoleaks	0 (0)	0 (0)	–
Type II endoleak	2 (2.9)	2 (4.7)	0.644
Type III endoleak	0 (0)	1 (2.3)	0.428
Type IV endoleak	2 (2.9)	2 (4.7)	0.644

Values are *n* (%) except that operation time, fluoroscopic time, blood loss and contrast volume are median (range). Operation time: duration between the beginning of vascular access exposure (surgically or percutaneously) and the completion of suture

Fluoroscopic time: duration between the first and the final digital subtraction angiography

*ISNF* in situ needle fenestration, *CS-Bp* carotid-subclavian bypass, *LSA* left subclavian artery

### Late Outcomes

**Fig. 4:**
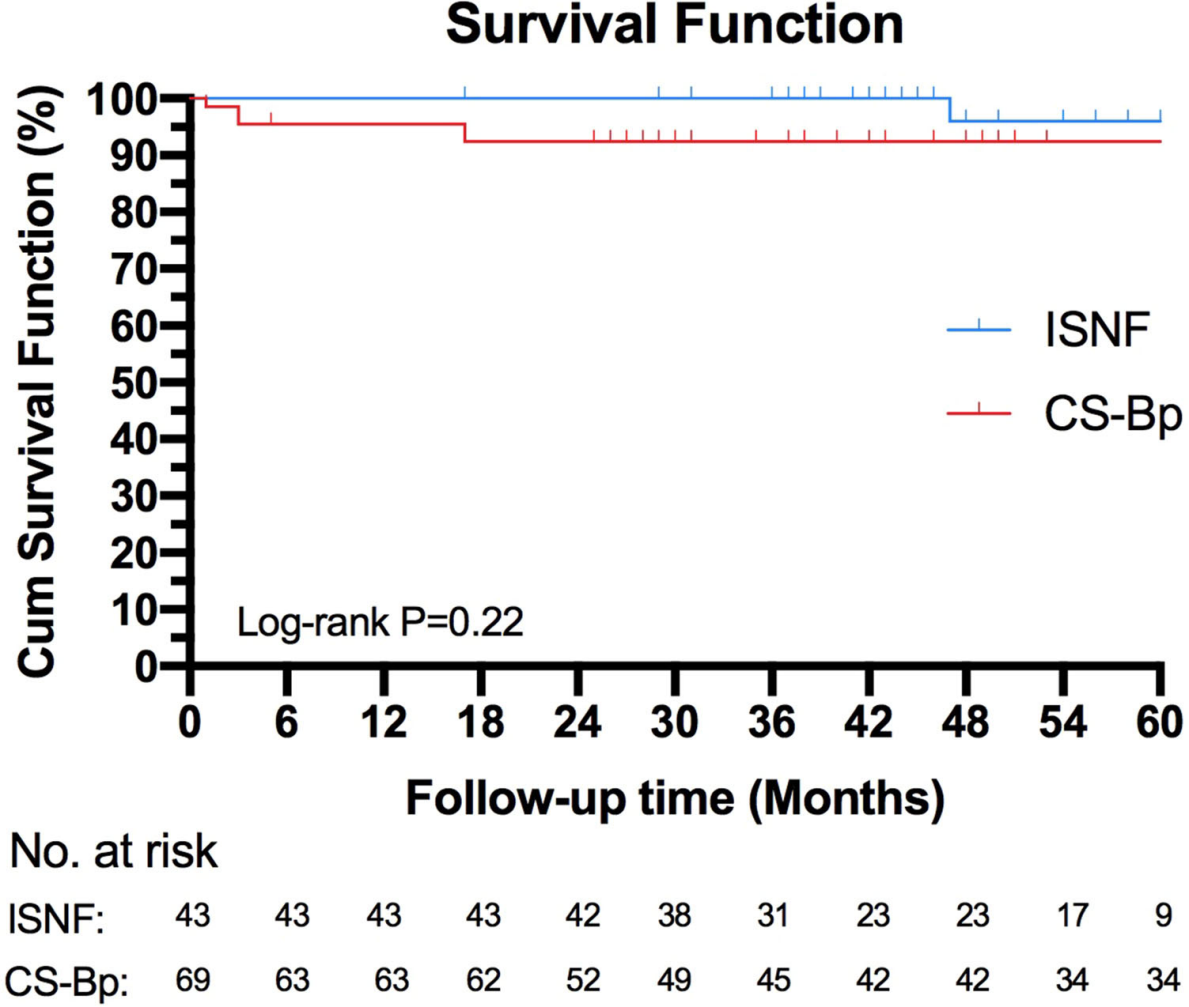
Kaplan–Meier estimates of overall survival

Late outcomes were assessed in 111 surviving patients (99.1%), with 108 of them (97.2%) available for follow-ups at median duration of 50 (maximum of 103) months. Six patients (5 in CS-Bp group and 1 in ISNF group) died during follow-ups, including one aortic-related (infection) in CS-BP group, three non-aortic-related (pulmonary embolism, gastrointestinal bleeding, cancer, severe pulmonary infection) and two unknown deaths where family members declined to provide information. The cumulative survival function was not significantly different between groups (ISNF 97.7% vs CS-Bp 89.9%, *p* = 0.22, Fig. 4). One patient in CS-Bp developed contralateral ischemia stroke in 3 months.

At the last available follow-up, the patency rates for the LSA and the left vertebral artery were 99.1% and 98.2%. One patient experiencing technical failure and conversion to chimney technique in the ISNF group received balloon angioplasty 6 months later due to LSA branch stenosis. Follow-ups of patients with endoleaks I–III are detailed in Table 4. All type IV endoleaks resolved during follow-ups. The aortic-related reintervention rate was 4.7% in the ISNF group, contrasting with zero occurrences in the bypass group.

**Table 4:** Details of patients with endoleaks or LSA occulsion

No	Group	Pathologies	Materials	Follow-ups
1	ISNF	Thoracic aortic aneurysms	Lifetech Ankura 38-30-200,7-37 Express LD	Type II endoleak (from the LSA) The estimated mean annual aortic grouth rate was 0.15 cm/year
2	ISNF	Type B aortic dissection	Lifetech Ankura 34-28-180, 8-25 Viabahn+ 7-50 Viabahn	Type II endoleak (from the LSA) The endoleak stoped 1 year later and achived complete descending aorta remodeling
3	ISNF	Type B aortic dissection	Lifetech Ankura 32-26-180, 8-50 Viabahn	Type III endoleak and received open surgery 6 months after TEVAR
4	ISNF	Type B aortic dissection	Lifetech Ankura 32-26-180, ISNF failed and convert to chimney technique (Fluency 7–40 mm)	LSA stenosis and received balloon angioplasty for the LSA 1 year later. The follow-up reveals a patent LSA with a bilateral blood pressure difference of 10 mmHg
5	CS-Bp	Thoracic aortic aneurysms	Medtronic 32-200, 10 mm maquet	Type II endoleak (from intercostal arterys) The estimated mean annual aortic grouth rate was 0.05 cm/year
6	CS-Bp	Type B aortic dissection	Medtronic 32-200, 8 mm maquet	Type II endoleak (from intercostal arterys) The estimated mean annual aortic grouth rate was 0.02 cm/year

*ISNF* in situ needle fenestration, *CS-Bp* carotid-subclavian bypass, *LSA* left subclavian artery

**Fig. 5:**
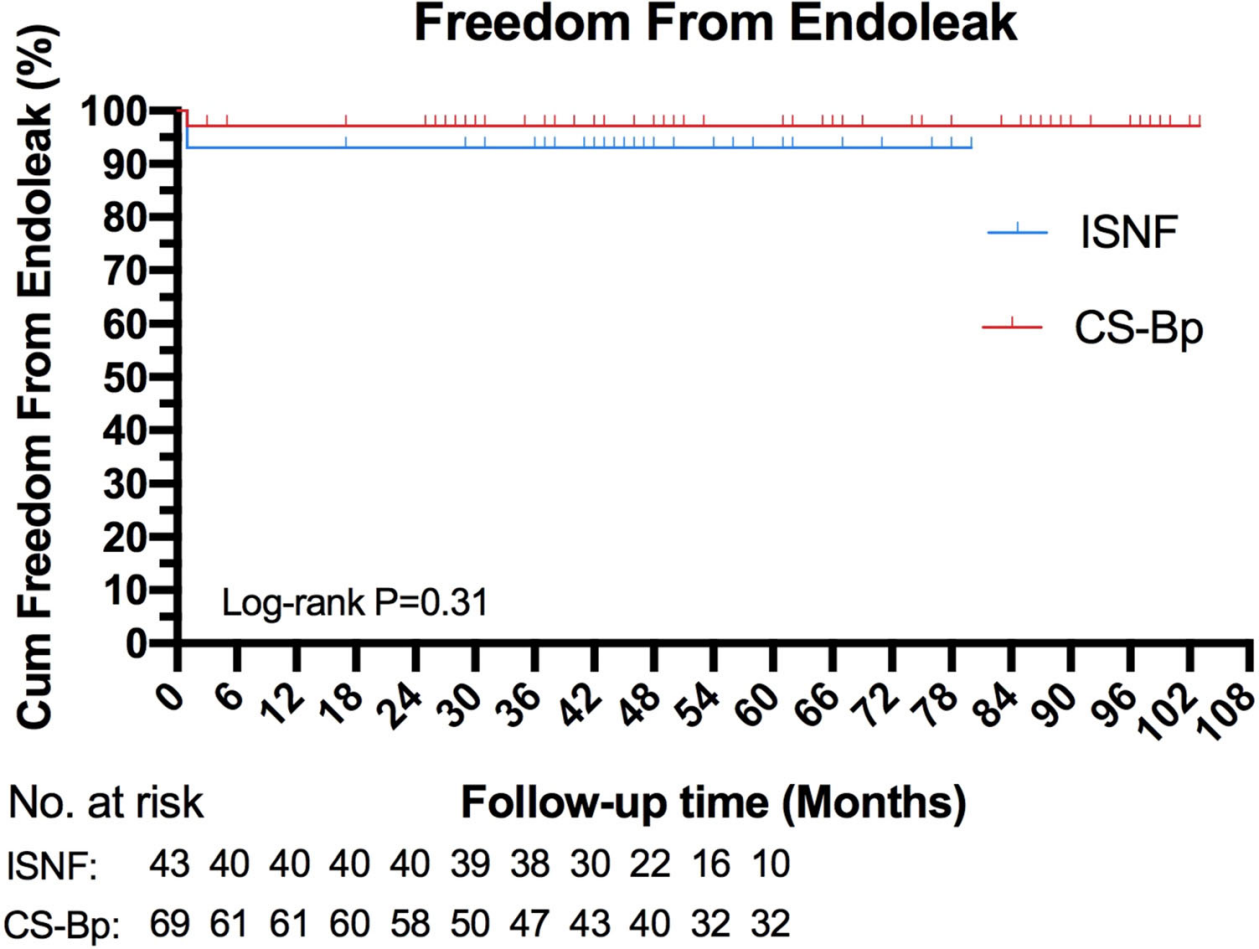
Cumulative freedom from endoleaks

The freedom from endoleak rates (including types I, II and III, CS-Bp 97.1% vs ISNF 93.0%) was not significantly different between the groups (*p* = 0.31, Fig. 5). Among different component combinations, there was no significant difference in terms of endoleak, either for the type of thoracic device or the LSA stent-graft.

## Discussion

Compared to CS-Bp technique, ISNF technique represents a less invasive total endovascular procedure to revascularize the LSA after TEVAR, allowing patients to experience a quicker recovery. Despite the ISNF group comprising patients of advanced age, with almost half having dissection, factors that could impact outcomes, it demonstrated an encouraging 0% perioperative complication rate, favorably contrasting with the 0–10% rates reported in recent years [16–21]. These positive results can be attributed to the following factors: Firstly, the application of TEVAR with ISNF does not require clamping of the LCCA, eliminating the need for LCCA ischemia. Additionally, the operation time of ISNF is notably shorter compared to surgical approaches. Second, the BPS and the Futhrough adjustable puncture needle ensures a stable position of the puncture system and allows for safe and accurate puncture procedures, as described above.

In the CS-Bp group, the perioperative mortality rate was 1.4%, and the stroke rate was 1.4%, aligning with the 0% to 5% mortality rates and the 0% to 5% stroke rates reported in the literature [4–7]. Both cases of postoperative paraplegia in the CS-Bp group were attributed to extended thoracic aortic stent-graft coverage. Despite prophylactic drainage of cerebrospinal fluid and management of systolic blood pressure, one patient unfortunately developed permanent paraplegia. The paraplegia rate was consistent with the 0%-10% reported in the literature [4–7, 16–21].

ISNF is an alternative approach and remains controversial [24]. Concerns about technical difficulties and vascular wall damages during the puncturing process of ISNF persist [16–21]. An experimental porcine study found no macroscopically visible emboli or clots after in situ laser fenestration, supporting the safety of the technique [25]. External studies indicate that different covering materials, when subjected to needle fenestration and laser fenestration, yield varied outcomes. Although not significant, it appears that laser puncture tends to weaken the materials in a greater way than needle fenestration [26]. Study Literature reviews demonstrated the feasibility of in situ fenestration, reporting a technical success rate ranging from 96% to 96.9% [27, 28]. Unlike CS-Bp which can be applied in most cases, patients planed for INSF are strictly selected by surgeons as described in the in/exclusion criteria for ISNF. The 4.7% type II endoleak rate and 2.3% type III endoleak rate in ISNF group align with the 0–8% endoleak rate reported in the literatures [16–21].

Several limitations should be acknowledged when interpreting the findings of this study. Firstly, the study employed a retrospective design, relying on existing medical records and data. This retrospective nature introduces the possibility of incomplete or missing data, potential inconsistencies in documentation and limited control over confounding variables. Secondly, the study sample was derived from a single institution, which may limit the generalizability of the findings to other settings or populations. Third, there may be some selection bias. Choice for CS-Bp and ISNF was based on the general condition of the patients and angio-anatomical criteria.

Despite these limitations, this study provides valuable insights into the comparative effectiveness of CS-Bp and ISNF with a relatively large sample size and mid-term follow-up results. Future research endeavors should address these limitations through prospective designs, larger sample sizes, multi-center collaborations, longer follow-up durations to further enhance the validity and generalizability of the findings.

## Conclusions

Both CS-Bp and ISNF are feasible techniques for reconstructing the LSA in TEVAR. ISNF appears to be fully competitive with CS-Bp. However, sufficient experience should be gained while selecting patients carefully. ISNF is considered an off-label technique and its use may impact the long-term durability of the stent-graft. Further improvements in equipment and techniques are necessary to enhance the reliability of ISNF, and data on long-term durability are essential before the technique is widely adopted.

## Abbreviations

TEVAR: Thoracic endovascular aortic repair
LCCA: Left common carotid artery
LSA: Left subclavian artery
DSA: Digital subtraction angiography
CS-Bp: Carotid-subclavian bypass
ISNF: In situ needle fenestration technique
BPS: Binding stent-graft puncture system
